# Constructing and evaluating ArabicStanceX: a social media dataset for Arabic stance detection

**DOI:** 10.3389/frai.2025.1615800

**Published:** 2025-06-19

**Authors:** Ali Alkhathlan, Faris Alahmadi, Faris Kateb, Hend Al-Khalifa

**Affiliations:** ^1^Computer Science Department, Faculty of Computing and Information Technology, King Abdulaziz University, Jeddah, Saudi Arabia; ^2^Information Technology Department, Faculty of Computing and Information Technology, King Abdulaziz University, Jeddah, Saudi Arabia; ^3^Information Technology Department, King Saudi University, Riyadh, Saudi Arabia

**Keywords:** stance detection, Arabic language, opinion mining, social media analysis, Arabic NLP

## Abstract

Arabic stance detection has attracted significant interest due to the growing importance of social media in shaping public opinion. However, the lack of comprehensive datasets has limited research progress in Arabic Natural Language Processing (NLP). To address this, we introduce ArabicStanceX, a novel and extensive Arabic stance detection dataset sourced from social media, comprising 14,477 tweets across 17 diverse topics. Utilizing the transformer-based MARBERTv2 model, we explore stance detection through Multi-Topic Single Model (MTSM) strategies, achieving a promising F1 score of 0.74 for detecting ‘favor' and ‘against' stances, and 0.67 overall. Our experiments highlight the model's capabilities and challenges, particularly in accurately classifying neutral stances and generalizing to unseen topics. Further investigations using zero-shot and few-shot learning demonstrate the model's adaptability to new contexts. This study significantly advances Arabic NLP, providing crucial resources and insights into stance detection methodologies and future research directions. The dataset is publicly available^1^.

## 1 Introduction

The digital era, marked by rapid technological advancements, constantly redefines our communication methods. New social media platforms emerge daily, promoting widespread connection and opinion sharing. Currently, over 58% of the global population uses social media, spending an average of 2–3 h online each day (Al Hendi, 2024).

A platform of significant interest to researchers is X.com (formerly Twitter), renowned for its ability to facilitate opinion expression. The diverse information within tweets provides valuable insights into public stance and behavior, fueling interest in “opinion mining” across fields such as Natural Language Processing (NLP) and social computing. The primary goal is to develop automated methods for measuring public opinion, supplementing traditional surveys.

Stance detection, a notable subfield of opinion mining, focuses on identifying whether an author's viewpoint in the text is supportive, opposing, or neutral toward a specific topic, such as an individual, legislation, or event. This task is crucial for applications like social media monitoring, opinion mining, and political analysis. For example, the tweet “Handguns should be banned in the US” illustrates a supportive stance on gun control.

With the proliferation of online platforms for sharing opinions, NLP research in stance detection has grown substantially. A pivotal development was the release of a stance detection dataset by Mohammad et al. (2016). Recent advancements in NLP and deep learning, particularly the development of transformer-based models like BERT (Bidirectional Encoder Representations from Transformers) (Devlin et al., 2019), have significantly enhanced stance detection capabilities. BERT's bidirectional fine-tuning approach allows it to understand the context of words within a sentence, making it highly effective for a wide range of NLP tasks.

Despite BERT's success in many languages, applying such models to Arabic text presents unique challenges due to the language's complex morphology, dialectal variations, and rich contextual semantics. Most stance detection research has focused on English due to the abundance of available datasets. However, other languages, like Arabic, have received less attention, with Arabic stance detection datasets being limited in terms of topic and diversity. This lack of comprehensive datasets represents a significant gap in NLP research.

This research aims to advance Arabic stance detection by introducing ArabicStanceX, a comprehensive and diverse dataset that can serve as a benchmark for a wide range of language models. To demonstrate its effectiveness, we evaluate it using MARBERTv2, a strong Arabic-specific baseline. It addresses the gap in available datasets by developing a comprehensive and diverse Arabic stance detection dataset from X.com tweets, called ArabicStanceX, focusing on Saudi Arabia due to its high X.com usage and active social media discussions. The number of X.com users in Saudi Arabia reached 5 million in 2012 and has since grown by 160%, reached ~13 million users by 2020 (Simsim, 2011). Addaitionally, recent legislation has sparked extensive discussions and debates among Saudis on social networks. While X.com is also widely used across other Arab countries, this study specifically focuses on Saudi Arabia due to both the platform's high penetration and the sociopolitical context that has triggered extensive public discourse in recent years. We acknowledge that this geographical focus may limit the generalizability of findings to other regions. However, the methodology and insights gained here lay the foundation for broader extensions to other Arabic-speaking communities.

This study introduces ArabicStanceX, an extensive dataset for Arabic stance detection comprising 14,477 instances across 17 topics, which will be publicly accessible to foster further research. It focuses on developing adaptable models for unseen topics using zero-shot and few-shot learning methodologies, evaluating various fine-tuning strategies with the MARBERTv2 model. The research investigates Single Topic Single Model (STSM) and Multi Topics Single Model (MTSM) approaches, enhancing MTSM with additional contextual information. Using *F*_*avg*2_ and *F*_*avg*3_ metrics, it assesses precision and recall for “favor” and “against” stances. Overall, the study makes significant contributions to Arabic NLP by providing a valuable dataset, exploring model adaptability, and evaluating effective fine-tuning and contextual strategies.

The rest of the paper is organized as follows: Section 2 reviews related work in stance detection, with a particular focus on previous datasets and methodologies. Section 3 details the methodology for developing the Arabic stance detection dataset, including data collection and annotation processes. Section 4 describes the experimental setup, including the BERT model, its hyperparameter tuning, and performance metrics. Section 5 presents the experimental results and their analysis. Finally, Section 6 concludes the paper and outlines promising directions for future research.

## 2 Related work and background

Stance detection research on social media platforms has gained significant traction in recent years. This research can be categorized into four main categories.

1. **Target-specific:** this category focuses on recognizing stances toward specific, predefined targets. For example, it identifies opinions related to particular issues like civil rights, where the stance is evaluated directly against a clearly defined subject.
2. **Multi-related targets:** in this approach, a single model is used to identify stances toward two or more interrelated subjects within the same text. For instance, the model might analyze the connection between civil rights and the death penalty, recognizing how opinions on one issue might influence or correlate with opinions on the other.
3. **Cross-target:** this category aims to develop classifiers that can transfer knowledge between various targets using a comprehensive dataset. The goal is to create models that are versatile and can apply learned stances from one target to different, previously unseen targets, thus enhancing the model's generalizability and adaptability.
4. **Target-independent:** this approach seeks to identify stances in comments related to news articles, focusing on tasks like confirming or denying the validity of the information or predicting whether different arguments support the same stance. This method does not rely on predefined targets but instead evaluates stances based on the context of the discussion.

These classifications help structure stance detection research, guiding the development of models and methods tailored to specific needs and applications in analyzing and understanding public opinions across various domains.

The field of stance detection received a significant boost with the launch of a shared task and the subsequent release of a publicly available dataset by Mohammad et al. (2016, 2017). This dataset, sourced primarily from X.com and focusing on predefined controversial topics like climate change and abortion, significantly increased research output compared to previous years (AlDayel and Magdy, 2021). Annotators on CrowdFlower categorized tweet-topic pairs into three stances: favor, against, or neutral.

Since then, additional stance detection datasets have emerged, catering to various domains. A substantial dataset of over 51,000 tweets focused on the financial domain was introduced in Conforti et al. (2020). The TW-BREXIT dataset, presented in Lai et al. (2020) contains 1,800 triplets of tweets related to the stance on leaving, remaining, or having no opinion on Brexit. Similarly, datasets addressing other controversial topics have been developed (Hosseinia et al., 2020; Grimminger and Klinger, 2021; Li et al., 2021; Gautam et al., 2020; Thakur and Kumar, 2021).

The investigation of stance detection has also expanded to include target-independent approaches, garnering considerable research interest. For instance, Gorrell et al. (2019) presented RumourEval, a claim-based dataset designed for stance classification within the context of rumors. This dataset covers a broad spectrum of events and categorizes tweets into four distinct stances: support, deny, query, or comment. Similarly, Hanselowski et al. (2018) proposed another dataset aimed at assessing stances toward various news headlines. These efforts are just a few examples, with additional datasets emerging in this vein by Ferreira and Vlachos (2016); Bar-Haim et al. (2017). Research has also explored cross-target stance detection (Allaway and McKeown, 2020; Vamvas and Sennrich, 2020; Kaur et al., 2016) and multi-target stance detection (Sobhani et al., 2017). Furthermore, efforts have been made to extend stance detection research to non-English languages, including Italian (Cignarella et al., 2020) and Spanish/Catalan (Taulé et al., 2017).

While stance detection datasets abound for English, Arabic resources remain scarce. A notable contribution is the fact-checking corpus by Baly et al. (2018), which links 402 Arabic claims to retrieved documents using a four-class stance scheme (agree, disagree, discuss, unrelated), annotated via crowdsourcing. While the dataset includes rationale spans for some labels, it is oriented toward long-form claim-document verification rather than general-purpose stance modeling. The Arabic News Stance corpus by Khouja (2020) comprises 3,786 claims, annotated through a multi-stage crowdsourcing process. It employs a three-class scheme (agree, contradict, other), merging “discuss” and “unrelated” into a single label to reduce ambiguity. While the dataset emphasizes real news headlines and achieves high inter-annotator agreement, it exhibits class imbalance and possible paraphrasing-induced variability.

AraStance (Alhindi et al., 2021) offers 4,063 claim–article pairs across multiple domains and Arab countries, labeled by graduate annotators using a four-class scheme (agree, disagree, discuss, unrelated). While its broad topical scope and refined annotation process enhance reliability, the dataset remains rooted in formal news sources and exhibits class imbalance. Expanding the options for Arabic stance detection, Alturayeif et al. (2022) introduced MAWQIF, a multi-dimensional dataset containing 4,121 Arabic tweets annotated for stance, sentiment, and sarcasm via Appen crowdsourcing. The stance labels follow a target-specific three-class scheme (favor, against, none), applied across three controversial topics. Although MAWQIF supports multi-task learning and includes dialectal variation, its coverage is limited to predefined targets, and it exhibits class imbalance due to low representation of neutral stances. Additionally, Jaziriyan et al. (2021) introduced EXaASC, a target-based stance dataset containing 9,566 Arabic tweet–reply pairs annotated by trained native speakers using a three-class scheme. With over 180 unique targets, it offers broad generalization potential, though its reply-based structure introduces conversational bias and a high proportion of none labels.

Table 1 summarizes these datasets, providing details on their name, language, stance type, text source, and size.

**Table 1:** Summary of stance detection datasets by name, language, source, and size.

**Name**	**Language**	**Stance type**	**Text source**	**Size**
SemEval2016-Task 6 (Mohammad et al., 2016, 2017)	English	Target specific	X.com	4,163 tweets
WT-WT (Conforti et al., 2020)	English	Target specific	X.com	51K
TW-BREXIT (Lai et al., 2020)	English	Target specific	X.com	1,800 triplets of tweets
Procon20 (Hosseinia et al., 2020)	English	Target specific	procon.org	6,094 of question and opinion
Hateful/offensive speech (Grimminger and Klinger, 2021)	English	Target specific	X.com	3K tweets
P-stance (Allaway and McKeown, 2020)	English	Target specific	X.com	21,574 tweets
MeTooMA (Gautam et al., 2020)	English	Target specific	X.com	9,973 tweets
RumourEval (Gorrell et al., 2019)	English	Target independent	X.com and Reddit	8,574 posts
FNC-1 (Hanselowski et al., 2018)	English	Target independent	News websites	75,385 instances and 2,587 news headlines
Emergent (Ferreira and Vlachos, 2016)	English	Target independent	Different websites	300 claims and 2,595 articles
IBM debater (Bar-Haim et al., 2017)	English	Target independent	Wikipedia	2,394 claims
Vast (Allaway and McKeown, 2020)	English	Cross target	News website	23,525 comments
X-stance (Vamvas and Sennrich, 2020)	Italian German French	Cross target	Smartvote.org	65 K
Multi-target SD (Sobhani et al., 2017)	English	Multi target	X.com	4,455 tweets
SardiStance (Cignarella et al., 2020)	Italian	Target Specific	X.com	3,242 tweets
IberEval (Taulé et al., 2017)	Spanish and Catalan	Target specific	X.com	11 K
Arabic fact checking (Baly et al., 2018)	Arabic	Target independent	Verify and Reuters	402 claims and 3,042 documents
Arabic news stance (Khouja, 2020)	Arabic	Target independent	News websites	3,786 pairs (claim, evidence)
AraStance (Alhindi et al., 2021)	Arabic	Target independent	Fact-checking websites	4,063 pairs of claim and article
MAWGIF (Alturayeif et al., 2022)	Arabic	Target specific	X.com	4,121 tweets
EXaASC (Jaziriyan et al., 2021)	Arabic	Cross-target	X.com	9,566 samples, and 180 targets

Research in stance detection has advanced significantly, but several notable gaps persist. Firstly, there is a scarcity of data in non-English languages, with most research focusing on English datasets. While efforts like AraStance and MAWGIF have contributed to Arabic resources, they remain more minor and less diverse compared to their English counterparts. Secondly, existing models often struggle with generalizability, especially when faced with unseen topics or targets. Cross-target stance detection methods aimed at enhancing adaptability to new targets with limited data are still in development. Additionally, current models primarily focus on explicit language, overlooking the role of context and implicit cues in sentence analysis. Elements like sarcasm and humor can be challenging for these models to interpret accurately.

To bridge these gaps, this study prioritizes creating more prominent and varied datasets in Arabic and other languages. Techniques like few-shot learning and domain adaptation have the potential to enhance model generalizability. Furthermore, incorporating contextual cues and sentence analysis can better capture the subtleties of human language. Through these efforts, stance detection can evolve into a more powerful tool for deciphering public opinion across diverse linguistic and cultural landscapes.

## 3 Methodology for ArabicStanceX dataset development

**Figure 1:**
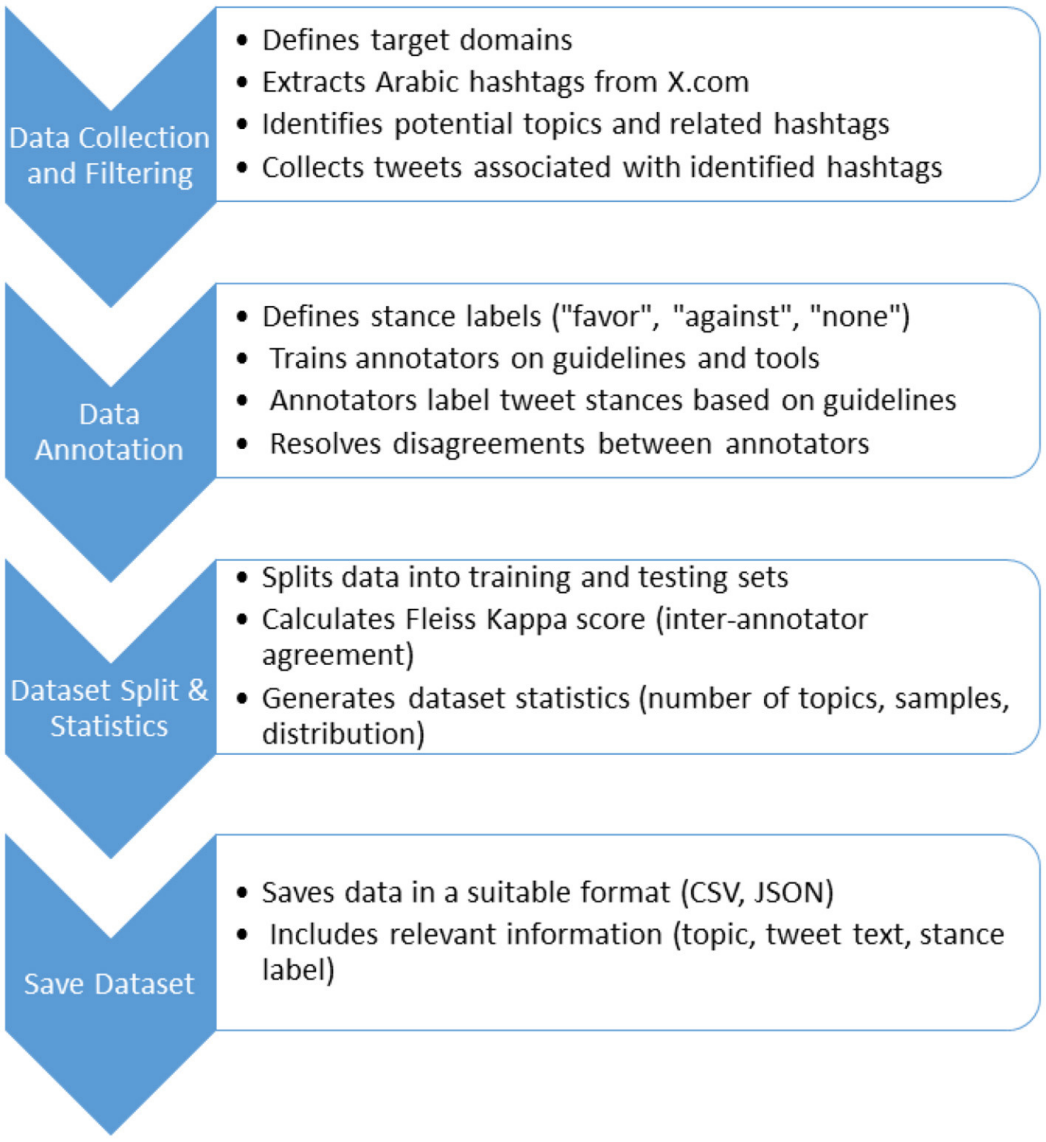
Methodology—ArabicStanceX dataset creation.

In this section, we detail the methodologies utilized in constructing the ArabicStanceX dataset. Our primary aim is to create a comprehensive, multi-topic dataset in Arabic that sets itself apart from previous datasets by offering extensive coverage and suitability for addressing novel targets, thus expanding its potential applications. Our research focused on data spanning from 2015 to 2021 in Saudi Arabia, a period marked by significant controversies. The dataset was sourced from X.com, making it currently the most exhaustive Arabic stance dataset available. The methodology for developing the Arabic stance detection dataset is illustrated in Figure 1 and described in the following subsections.

### 3.1 Data collection and filtering

Our initial step was to create a collection of pre-defined, controversial topics that would elicit strong opinions. We achieved this by first extracting all hashtags from X.com within Saudi Arabia between 2015 and 2021. We then analyzed these hashtags to identify potential topics. Specifically, we manually reviewed the most frequently occurring hashtags and selected those that were associated with real-world events, public policies, or debates that sparked polarized public engagement. Hashtags were grouped into candidate topics if they reflected a clearly defined issue with both supportive and opposing discourse. Once a topic was identified, we used its relevant keywords to find all related hashtags, ensuring a broad spectrum of areas like sports, economy, education, health, religion, and culture (details in Table 1).

To capture a diverse range of viewpoints, we collected hashtags representing both supportive and opposing stances for each topic. For instance, on the topic of women driving, we included hashtags like “#WomenShouldDrive” and “#WomenShouldNotBeDriving.” This approach ensured we captured a spectrum of opinions, from agreement to disagreement.

After collecting the data, we organized it into distinct domains, each containing specific topics with their associated hashtags and tweets. We then performed several preprocessing steps:

1. **Language filtering:** we filtered out all non-Arabic tweets, keeping only Arabic content.
2. **Noise removal:** we removed retweets, user mentions, URLs, and duplicate tweets. To identify subtle duplicates, we employed SentenceTransformer “paraphrase-xlm-r-multilingual-v1” by Reimers and Gurevych (2019) to measure tweet similarity. Tweets with a cosine similarity exceeding 0.95 were discarded.
3. **Advertisement removal:** analysis of a random sample of 1,000 tweets revealed that tweets with four or more hashtags were predominantly advertisements. Consequently, we eliminated all such tweets from the dataset.

Table 2 provides a list of the domains and their associated target topics.

**Table 2:** Details of the specific domains and their related topics.

**Domain**	**Topic**	**Topic description**
Economy	Aramco Share Selling	Aramco made available a part of their total company shares, amounting to 1.5%, for trading among the general public.
	Al-Qiddiya Project	Al-Qiddiya is a Saudi sport, cultural, and entertainment project which will be located in the city of Al-Qiddiya, which serves as a high-quality entertainment and social destination.
	Neom City	The Kingdom of Saudi Arabia has planned to construct a novel urban district, Neom, in the northwestern Tabuk Province.
Education	Teaching Chinese Language at School	The Saudi Ministry of Education has announced to include Chinese language in the curriculum of Saudi public schools.
	Improve School Curriculum	The Saudi Ministry of Education unveiled a new educational system and curriculum that comprises new subjects and a reduction in the number of classes for religious studies.
	Online Learning	Transitioning from conventional to online teaching during COVID-19
Health	COVID-19 Vaccine	The Saudi authorities are mandating that Saudi citizens receive the COVID-19 vaccine.
	Vaccine Booster Dose	The Saudi authorities are mandating that Saudi citizens receive the COVID-19 booster dose.
Sports	Prince Abdulaziz bin Turki Head of Sports Minister	Appointing Prince Abdulaziz bin Turki as a minister of sports.
	Prince Faisal bin Turki as Resignation from a Saudi club	Prince Faisal bin Turki as resignation from Al-nasser Saudi club.
Religion/ Cultural	Sex Education	Implementing a sex education curriculum in Saudi public school.
	Coexistence with Religions	The peaceful coexistence and dialogue among religions.
	Women driving	Allowing women to drive in Saudi Arabia.
	Mosques Speakers	Limiting the utilization of mosque loudspeakers exclusively for the Adhan (the call to prayer) while retaining their use within the mosque premises during prayer times.
	Polygamous marriage	Deciding whether to endorse the concept of simultaneous multiple spouses.
Other	Domestic tourism	Supporting domestic tourism in the Kingdom of Saudi Arabia
	Military conscription	The mandatory enlistment of Saudi citizens in the armed forces

### 3.2 Data annotations

To ensure the accuracy of our stance labels, we partnered with Wosom, a Saudi company staffed with native Arabic speakers (Wosom, 2024). Wosom took on the responsibility of both conducting the annotations and upholding high-quality standards throughout the process.

Before embarking on the main annotation task, we initiated a pilot test using a smaller subset of the data. The purpose of this pilot test was to confirm the clarity of our annotation guidelines and validate the functionality of the annotation tools. We conducted the pilot test through multiple iterations, reviewing a random sample of 50 tweets from various topics after each iteration to identify and address any potential issues.

Three native Saudi speakers were meticulously selected based on their language proficiency, attention to detail, and relevant domain expertise to annotate each tweet. Subsequently, these annotators underwent rigorous training on the annotation guidelines and the Wosom annotation platform. They were provided with clear instructions and relevant examples to ensure the accuracy of their annotations. Throughout the annotation process, continuous feedback from reviewers and validators was incorporated to maintain high-quality standards. Each of the 14,477 tweets was independently annotated by all three annotators to ensure consistent labeling and enable majority agreement.

In instances of disagreement regarding the classification of a tweet, an adjudication method was implemented. This involved applying established criteria or engaging in group discussions facilitated by a designated team member to reach a consensus.

The annotators categorized tweets related to each topic into three distinct categories: “favor,” “against,” or “none.” Tweets expressing explicit or implicit support for the topic were labeled as “favor,” while those opposing the topic in either direct or indirect ways were labeled as “against.” Tweets that did not express a stance or were unrelated to the topic, such as advertisements, were categorized as “none.”

### 3.3 Dataset statistics

The ArabicStanceX dataset comprises 17 distinct topics with a total of 14,477 samples. To gauge the agreement between annotators, we computed an average Fleiss Kappa score of 0.54 across all topics. Subsequently, we partitioned the dataset into training and testing sets, utilizing an 80:20 split for model development and evaluation. Detailed statistics for individual topics within both sets are presented in Table 3.

**Table 3:** Data statistics for each label across all topics, segmented into the training and testing sets.

**Domain**	**Topics**	**# Training samples (80%)**				**# Testing samples (20%)**				**Total samples**
		**Favor**	**Against**	**None**	**Total**	**Favor**	**Against**	**None**	**Total**	
Education	Teaching Chinese language at school	336	297	65	698	85	75	17	177	875
	Improve School Curriculum	308	390	87	785	77	98	22	197	982
	Online Learning	297	326	111	734	75	82	28	185	919
Health	COVID-19 Vaccine	330	361	46	737	83	91	12	186	923
	COVID-19 Vaccine Booster Dose	280	372	105	757	70	93	27	190	947
Economy	Aramco Share Selling	297	293	132	722	75	74	34	183	905
	Al-Qiddiya Project	500	128	80	708	125	32	21	178	886
	Neom City	406	193	133	732	102	49	34	185	917
Other	Domestic Tourism	340	183	216	739	85	46	54	185	924
	Military Conscription	328	324	106	758	82	81	27	190	948
Sport	Prince Abdulaziz bin Turki Head of Sports Minister	63	72	100	235	16	18	60	94	329
	Prince Faisal bin Turki's Resignation from a Saudi club	100	61	123	284	70	16	31	117	401
Religion/ Culture	Women Driving	372	268	116	756	93	68	30	191	947
	Mosques Speakers	140	428	106	674	35	107	27	169	843
	Polygamous marriage	306	252	112	670	77	64	28	169	839
	Sex education	324	336	113	773	81	84	29	194	967
	Coexistence with religions	253	168	317	738	64	43	80	187	925
	Total	4980	4452	2068	11500	1295	1121	561	2977	14477

**Figure 2:**
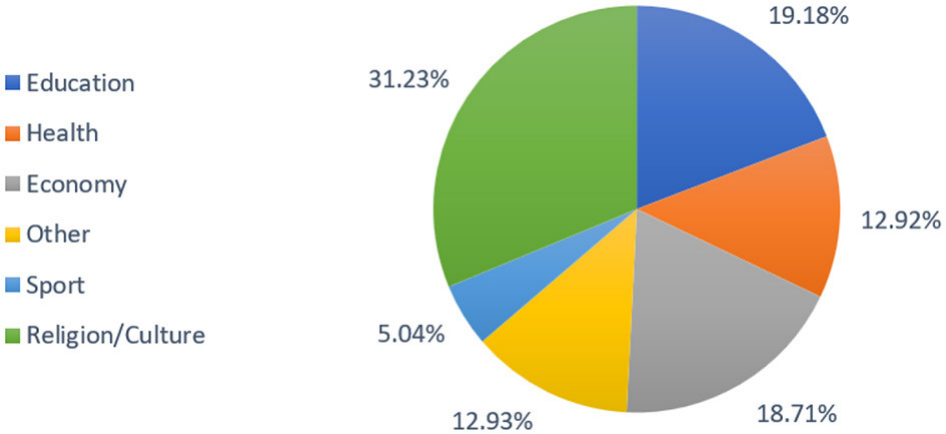
Distribution of samples across dataset domains.

Figure 2 illustrates the distribution of topics within the dataset, with a predominant focus on Religion/Culture (31.2%), followed by Education (19.1%), Economy (18.7%), Other (12.9%), and Health (12.9%). Sports constitute the most minor portion at 5.04%.

**Figure 3:**
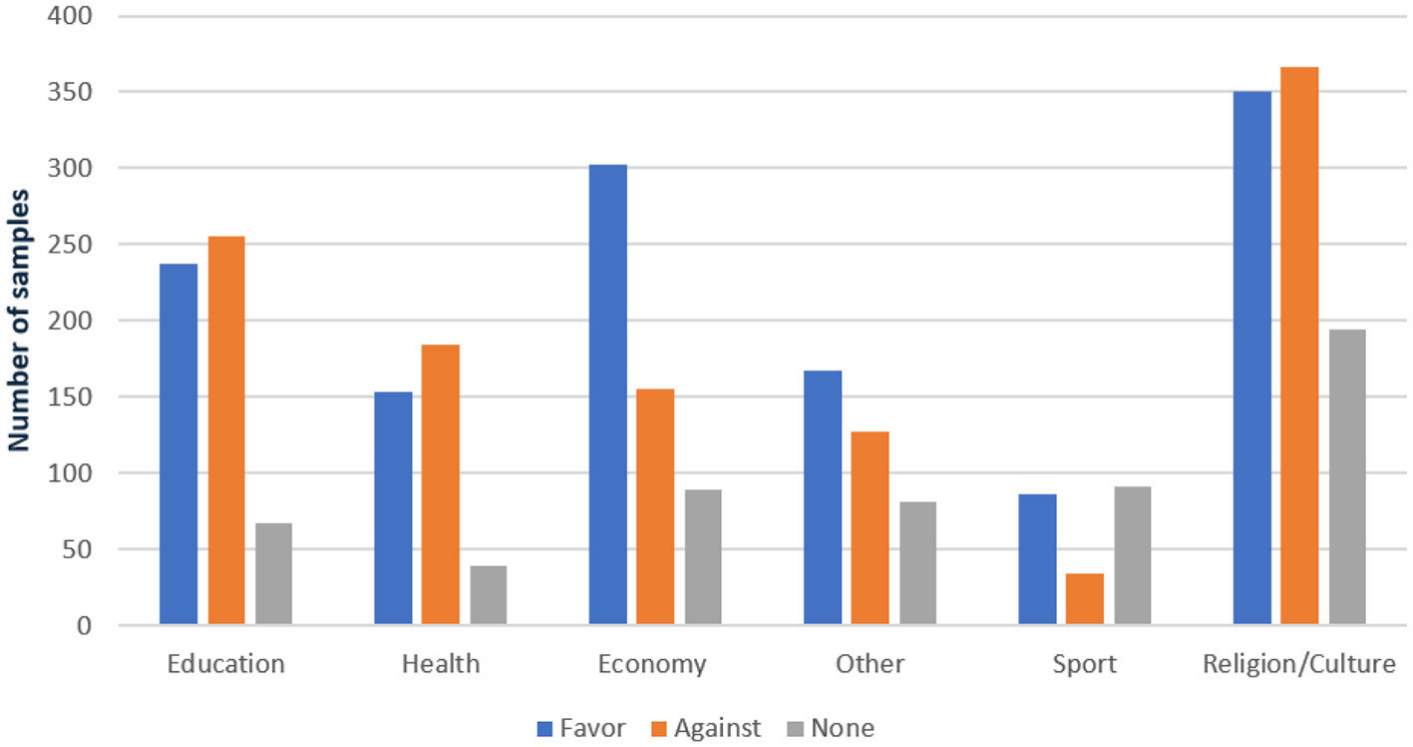
Distribution of class labels for training and testing sets across domains in the dataset.

Further granularity is provided in Figure 3, which delineates the distribution of training and testing samples across these domains. This meticulously organized structure underscores the dataset's diversity and its coverage of a wide array of topics. Such diversity lays a robust groundwork for conducting thorough analyses and developing resilient Arabic stance detection models. The structured approach facilitates nuanced research and model training, thereby contributing to advancements in Arabic computational linguistics.

## 4 Experimental setup

In evaluating the efficacy of the ArabicStanceX dataset, we harnessed the power of the BERT (Bidirectional Encoder Representations from Transformers) architecture across different contexts. This section provides insights into BERT and the particular models we utilized for assessment. Additionally, we delve into the experimental configuration, encompassing hyperparameter adjustments, and elucidate the performance metrics employed to measure the effectiveness of the models.

### 4.1 Model selection

This research leverages the power of Bidirectional Encoder Representations from Transformers (BERT) as the cornerstone of the ArabicStanceX dataset model. Developed by Google AI, BERT stands out for its exceptional ability to grasp the intricate relationships between words within a sentence (Devlin et al., 2018). Unlike traditional models that process text word by word, BERT employs a bidirectional approach. It analyzes both the preceding and following words, enabling it to capture the subtle nuances of language with remarkable precision. This bidirectional processing allows BERT to unlock the more profound meaning inherent in the text. By pre-training on massive amounts of text data, BERT learns to encode rich contextual information. This empowers it to excel in various Natural Language Processing (NLP) tasks, including sentiment analysis, text classification, and question answering.

In the realm of stance detection, where understanding an author's sentiment toward a topic is crucial, BERT's bidirectional processing proves invaluable. It delves into the full context of an Arabic sentence to discern whether the author's stance is supportive, opposing, or neutral regarding the embedded topic. However, to harness BERT's full potential for Arabic stance detection, fine-tuning is essential. This process involves adjusting BERT's internal parameters specifically for this task. Essentially, we train BERT to recognize the subtle ways in which stance is expressed within Arabic text. Through fine-tuning, BERT becomes adept at navigating the nuances of the Arabic language, offering valuable insights into public opinion and sentence across diverse topics and discussions.

**Figure 4:**
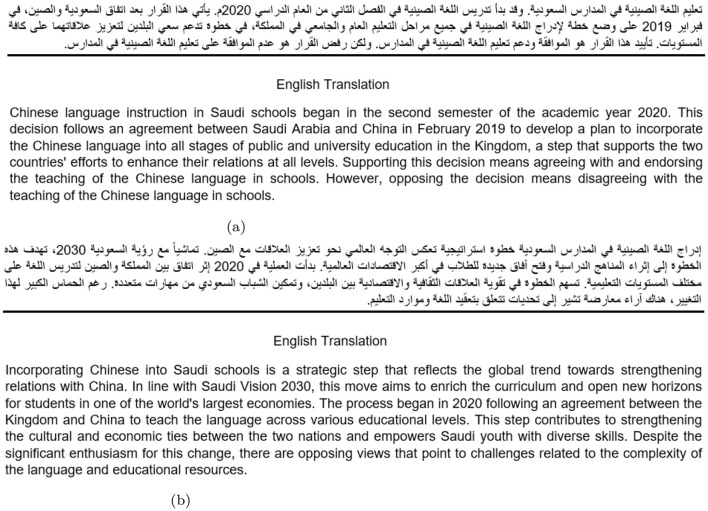
Example of manually crafted and ChatGPT generation of topic description for the topic of teaching Chinese language in Saudi schools. **(a)** Manually crafted topic description. **(b)** opic description generation by ChatGPT-GPT4.

We investigate different approaches for fine-tuning BERT during this phase, as outlined below:

1. **Single Topic Single Model (STSM):** in the STSM strategy, we employ a single input BERT structure. Initially, our focus was on fine-tuning a dedicated BERT-based model for each specific topic. This involved adjusting the weights of the pre-trained model to understand better the overall context and unique characteristics of each topic. The objective was to develop specialized models tailored to individual subject areas. However, we ultimately reconsidered this approach due to its consistent failure to capture the “None” stance across various topics effectively. This limitation revealed challenges in generalizing the models and accurately representing less common classes within single-topic analysis.
2. **Multi Topics Single Model (MTSM):** in the MTSM approach, we simultaneously fine-tune a single BERT-based model across all topics. This method allows the model to learn from a diverse range of subject matters in a unified manner, potentially improving its ability to discern commonalities and differences among topics. By fine-tuning the model on a broader dataset, we aim to enhance its generalization capabilities and its proficiency in handling multiple topics within a single framework. MTSM involves fine-tuning a combined dataset with variations in input data structure:

- **MTSM-None:** this model utilizes a single input sequence BERT architecture, fine-tuning the language model based solely on the tweet content without additional contextual information. The aim is to evaluate the model's stance inference capability from tweet text alone.
- **MTSM-Keywords:** employing a two-input-sequence BERT architecture, this method incorporates topic-specific keywords along with the tweets during fine-tuning. Including keywords aims to enhance the model's sensitivity to topic-specific nuances.
- **MTSM-Topic Description:** to ensure the model adequately captures topic-related nuances, we explore two strategies for providing it with sufficient topic description. The first strategy involves manually crafting a template-based description for each topic, guiding the content of the descriptions. The second strategy leverages GPT-4-ChatGPT to automatically generate relevant descriptions for each topic, potentially increasing scalability. An example of MTSM-Topic Description for teaching Chinese language in Saudi schools is provided in Figure 4.

### 4.2 Experimental design

This section elucidates the specific variant of the BERT model employed in our study, the process of hyperparameter tuning, and the performance metrics utilized for evaluation.

#### 4.2.1 BERT model used

In this study, we employed the MARBERTv2 model, renowned for its exceptional performance in handling various Arabic dialectal tasks (Elmadany et al., 2022). The selection of MARBERTv2 was motivated by its state-of-the-art capabilities in comprehending and processing the intricacies of Arabic dialects, rendering it particularly well-suited for our stance detection task across a wide array of topics sourced from social media data. MARBERTv2 was fine-tuned on our dataset, as outlined in the Model section, utilizing the Multi Topics Single Model (MTSM) approach simultaneously across all topics. Additionally, we experimented with both single and two-input BERT architectures. In all our methodologies, we utilized the BERT [CLS] token as the text representation embedding of the input text.

#### 4.2.2 Hyperparameters tuning

In optimizing the hyperparameters for the MARBERTv2 model, our strategy aimed to fine-tune the settings to improve both fine-tuning efficiency and model performance. We employed the AdamW optimizer (Kingma and Ba, 2014), renowned for its effectiveness in handling sparse gradients on noisy problems. Our experiments utilized a constant learning rate of 2e-5, supplemented by beta coefficients of 0.9 and 0.999, and an epsilon value of 1e-8 to ensure robust convergence. To prevent overfitting, the model underwent a weight decay of 0.001 and employed a dropout rate of 0.1. The fine-tuning spanned 25 epochs with a batch size of 32. Input sequences were restricted to 128 tokens for single inputs and extended to 512 for composite inputs involving topics, balancing computational resources with comprehensive contextual understanding.

#### 4.2.3 Evaluation metrics

Our evaluation of the baseline models centers on two specialized metrics: *F*_*avg*2_ and *F*_*avg*3_ scores. The *F*_*avg*2_ score represents a macro-average F1 score tailored for the “favor” and “against” stance labels, deliberately excluding the “none” class due to its minimal presence in our dataset. The *F*_*avg*2_ score is computed using Equation 1.


(1) $$\begin{array}{l} F_{avg2}=\frac{F_{favor}+F_{against}}{2} \end{array}$$

Here, *F*_*favor*_ and *F*_*against*_ represent the F1 scores for the “favor” and “against” classes, respectively. These scores are derived from the precision and recall of each class as per Equations 2–3.


(2) $$\begin{array}{l} Precision=\frac{TP}{TP+FP} \end{array}$$

(3) $$\begin{array}{l} Recall=\frac{TP}{TP+FN} \end{array}$$

We opted for the *F*_*avg*2_ metric to ensure alignment with other stance detection studies that report their findings using the same metric (Mohammad et al., 2016).

In addition to *F*_*avg*2_, we present results using the *F*_*avg*3_ metric, which accounts for all stance labels, including “none". The *F*_*avg*3_ score represents an average of the F1 scores for all three stances and is calculated as per Equation 4.


(4) $$\begin{array}{l} F_{avg3}=\frac{F_{favor}+F_{against}+F_{none}}{3} \end{array}$$

By reporting both *F*_*avg*2_ and *F*_*avg*3_ scores, our evaluation provides a comprehensive reflection of the model's performance in stance detection, encompassing both specific and overall detection capabilities.

## 5 Experiments and result analysis

We assessed the efficacy of the ArabicStanceX dataset, MARBERTv2, an Arabic Language Model, for stance detection across a range of topics. Our evaluations encompassed various fine-tuning approaches within the MTSM framework, including scenarios involving few-shot learning. Performance of different methods was gauged based on the ArabicStanceX dataset using performance metrics outlined in Section 4.2.3.

### 5.1 Performance analysis of MTSM model

We performed a series of experiments using the MTSM model with the ArabicStanceX dataset. The results are showcased in Table 4 employing the MTSM-None approach. In this experimental setup, the model fine-tunes a BERT-based language model solely on tweets without supplementary context, leading to notable performance variations across different topics. For example, the model achieves high F1 scores for “favor” and “against” classes in education-related topics like “Teaching Chinese Language at School.” However, scores are notably lower for topics involving specific individuals, such as “Prince Abdulaziz bin Turki, Head of Sports Minister,” suggesting challenges in stance detection when the input lacks contextual cues. The average F1 scores indicate that while the model performs adequately in some areas, it struggles in contexts requiring a deeper understanding of sentence, as evidenced by lower scores in complex social topics.

**Table 4:** F1 scores for “favor,” “against,” and “none” stances using MTSM-None).

**Topic**	***F*_*favor*_**	***F*_*against*_**	***F*_*none*_**	***F*_*avg*2_**	***F*_*avg*3_**
Teaching Chinese Language at School	0.90	0.91	0.46	0.90	0.75
Improve School Curriculum	0.91	0.887	0.40	0.89	0.73
Online learning	0.88	0.88	0.67	0.88	0.81
COVID-19 Vaccine	0.80	0.80	0.31	0.80	0.64
COVID-19 Vaccine Booster Dose	0.82	0.77	0.54	0.79	0.71
Aramco Share Selling	0.84	0.89	0.66	0.87	0.80
Al-Qiddiya Project	0.89	0.63	0.41	0.76	0.64
Neom City	0.90	0.79	0.65	0.84	0.78
Domestic Tourism	0.74	0.67	0.52	0.70	0.64
Sex Education	0.75	0.75	0.54	0.75	0.68
Coexistence with Religions	0.60	0.51	0.66	0.56	0.59
Military Conscription	0.76	0.77	0.64	0.77	0.73
Prince Abdulaziz bin Turki Head of Sports Minister	0.40	0.51	0.63	0.45	0.51
Prince Faisal bin Turki as Resignation from a Saudi club	0.48	0.36	0.45	0.42	0.43
Women_Driving	0.79	0.69	0.45	0.74	0.65
Mosques Speakers	0.57	0.76	0.24	0.67	0.53
Polygamous Marriage	0.83	0.83	0.49	0.83	0.71
AVERAGE OVER Avg2 & Avg3				0.74	0.66

Table 4 shows the performance of the MTSM-None approach, which uses BERT to classify stances based solely on the tweet content for various topics in the dataset. The table includes F1 scores for three categories: “favor,” “against,” and “none.” The F1 score is a metric that balances precision (accuracy of identifications) and recall (completeness of identifying positive cases). The obtained results are explained below.

1. Overall performance: the average F1 score across all topics considering both “favor” and “against” stances (*F*_*avg*2_) is 0.74, with an average considering all three stances (*F*_*avg*3_) being 0.66. This indicates that the model performs moderately well in stance detection using only tweet content.
2. Topic-wise performance: the performance varies depending on the topic. Some topics like “Teaching Chinese Language at School” and “Aramco Share Selling” achieved high F1 scores for both “favor” and “against” stances (above 0.9 for *F*_*avg*2_). This suggests the model can effectively classify tweets expressing explicit opinions on these topics.
3. Neutral stance (“none") classification: the model struggles with identifying neutral stances (“none") across most topics. This is evident from the consistently lower F1 scores for “none” compared to “favor” and “against.” Topics like “Coexistence with Religions” and “Mosques Speakers” show particularly low scores for “none,” indicating difficulty in distinguishing neutral tweets from those expressing an opinion on these sensitive subjects.

Overall, the results suggest that the MTSM-None approach achieves reasonable performance in stance detection for some topics with explicit opinions expressed in the tweets. However, the model has limitations in identifying neutral stances, especially for sensitive or complex topics. This highlights the potential need for incorporating additional information beyond just tweet content, such as topic descriptions or keywords, to improve the model's ability to handle diverse stances and topics.

Table 5 shows the performance of the MTSM-Keywords fine-tuning approach for stance detection on various Arabic topics. Each row represents a specific topic identified by its keywords. The columns “*F*_*favor*_,” “*F*_*against*_,” and “*F*_*none*_” present the F1 scores, a metric used to evaluate model performance, for tweets classified as “favor,” “against,” and “none” stances on that topic, respectively. The “*F*_*avg*2_” and “*F*_*avg*3_” columns represent the average F1 scores across two different evaluation methods (potentially macro and micro averaging). Looking at the average scores at the bottom of the table (AVERAGE OVER Avg2 & Avg3), we see that the model performs moderately well overall, with an average F1 score of 0.72 for identifying tweets expressing a stance (“favor” or “against") and 0.66 for classifying tweets with a neutral stance (“none"). However, the performance varies across topics. Some topics, like “Online Learning” and “Aramco Share Selling,” achieved high F1 scores for all stances, indicating the model's ability to classify tweets related to these topics accurately. Conversely, topics like “Coexistence with Religions” and “Prince Faisal bin Turki's Resignation” resulted in lower F1 scores, suggesting the model struggled to distinguish stances on these subjects. It's important to note that some topics might be inherently more challenging due to the nature of the discussion. For instance, “Coexistence with Religions” might involve a wider range of nuanced opinions that are difficult to categorize definitively as “favor” or “against.” Overall, the results suggest that the MTSM-Keywords approach offers a promising foundation for stance detection in Arabic text. However, further investigation might be needed to improve performance on specific topics.

**Table 5:** F1 scores for “favor,” “against,” and “none” stances using MTSM-Keywords.

**Topic keywords**	**With topic keywords**				
	*F*_*favor*_	*F*_*against*_	*F*_*none*_	*F*_*avg*2_	*F*_*avg*3_
Teaching Chinese Language at School	0.9	0.91	0.62	0.9	0.81
Improve School Curriculum	0.79	0.83	0.43	0.81	0.68
Online Learning	0.92	0.91	0.73	0.91	0.85
COVID-19 Vaccine	0.83	0.8	0.29	0.82	0.64
COVID-19 Vaccine Booster Dose	0.82	0.84	0.59	0.83	0.75
Aramco Share Selling	0.81	0.88	0.64	0.85	0.78
Al-Qiddiya Project	0.88	0.67	0.5	0.78	0.68
Neom City	0.89	0.75	0.66	0.82	0.77
Domestic Tourism	0.64	0.57	0.49	0.61	0.57
Sex Education	0.72	0.8	0.56	0.76	0.69
Coexistence with Religions	0.44	0.44	0.63	0.44	0.5
Military Conscription	0.72	0.7	0.56	0.71	0.66
Prince Abdulaziz bin Turki Head of Sports Minister	0.27	0.43	0.68	0.35	0.46
Prince Faisal bin Turki as Resignation from a Saudi club	0.43	0.44	0.44	0.44	0.44
Women_Driving	0.77	0.68	0.55	0.72	0.67
Mosques Speakers	0.55	0.78	0.33	0.67	0.56
Polygamous Marriage	0.84	0.84	0.59	0.84	0.76
AVERAGE OVER Avg2 & Avg3				0.72	0.66

Table 6 shows the results (F1 scores) for the MTSM (Multi-Topic, Single Model) approach with two different topic descriptions: manually crafted and generated by GPT-4. F1 score is a metric that balances precision and recall, providing an overall measure of model performance. Looking across the table, we see that both topic description methods achieved similar performance on average. The average F1 score for both “favor” and “against” stances is around 0.8 for both manual and GPT-4 descriptions, indicating good model performance in identifying supportive and opposing opinions. However, the results for the “none” stance, which represents tweets that don't express a clear opinion, are lower. The average F1 score for “none” is around 0.5 for both methods, suggesting more difficulty in accurately classifying neutral tweets.

**Table 6:** F1 scores for “favor,” “against,” and “none” stances using MTSM as topic description.

**Topic**	**Manual topic description**					**GPT4 topic description**				
	*F*_*favor*_	*F*_*against*_	*F*_*none*_	*F*_*avg*2_	*F*_*avg*3_	*F*_*favor*_	*F*_*against*_	*F*_*none*_	*F*_*avg*2_	*F*_*avg*3_
Teaching Chinese Language at School	0.90	0.91	0.47	0.91	0.76	0.88	0.91	0.53	0.90	0.77
Improve School Curriculum	0.88	0.90	0.48	0.89	0.75	0.85	0.87	0.44	0.86	0.72
Online Learning	0.91	0.88	0.71	0.89	0.83	0.84	0.83	0.62	0.84	0.76
COVID-19 Vaccine	0.81	0.81	0.23	0.81	0.62	0.81	0.77	0.28	0.79	0.62
COVID-19 Vaccine Booster Dose	0.84	0.82	0.61	0.83	0.76	0.77	0.78	0.52	0.77	0.69
Aramco Share Selling	0.84	0.88	0.61	0.86	0.78	0.85	0.88	0.59	0.87	0.78
Al-Qiddiya Project	0.90	0.68	0.47	0.79	0.68	0.88	0.65	0.40	0.76	0.64
Neom City	0.90	0.75	0.71	0.83	0.79	0.90	0.78	0.67	0.84	0.78
Domestic Tourism	0.71	0.62	0.54	0.66	0.62	0.72	0.67	0.54	0.70	0.64
Sex Education	0.76	0.73	0.52	0.75	0.67	0.70	0.67	0.55	0.68	0.64
Coexistence with Religions	0.61	0.39	0.66	0.50	0.55	0.60	0.41	0.65	0.51	0.56
Military Conscription	0.77	0.76	0.57	0.77	0.70	0.71	0.67	0.64	0.69	0.67
Prince Abdulaziz bin Turki Head of Sports Minister	0.46	0.47	0.72	0.47	0.55	0.43	0.33	0.58	0.38	0.45
Prince Faisal bin Turki as Resignation from a Saudi club	0.43	0.43	0.50	0.43	0.46	0.58	0.20	0.39	0.39	0.39
Women_Driving	0.78	0.62	0.51	0.70	0.64	0.78	0.67	0.56	0.72	0.67
Mosques Speakers	0.48	0.76	0.33	0.62	0.52	0.54	0.78	0.36	0.66	0.56
Polygamous Marriage	0.80	0.85	0.53	0.82	0.73	0.83	0.84	0.45	0.84	0.71
AVERAGE OVER Avg2 & Avg3				0.74	0.67				0.72	0.65

There are some interesting variations between topics. For instance, both methods performed well on topics like “Teaching Chinese Language at School” and “Aramco Share Selling,” achieving high F1 scores across all stances. Conversely, topics like “Coexistence with Religions” and “Mosques Speakers” proved more challenging, with lower F1 scores especially for the “none” stance. This suggests that these topics might be more nuanced or have a higher prevalence of neutral language, making stance detection more difficult. Overall, the results indicate that the MTSM approach with either manually crafted or GPT-4 generated topic descriptions can effectively identify supportive and opposing stances in Arabic text for a variety of topics. However, there's room for improvement in accurately classifying neutral tweets, and some topics may require further investigation or model improvements for better performance.

### 5.2 Performance analysis of few-shot learning model

This section explores the effectiveness of ArabicStanceX dataset in real-world situations where it might encounter entirely new topics, which were unseen during fine-tuning. This is particularly relevant for stance detection as new topics frequently emerge and quickly capture public attention. To address this challenge, we employed few-shot learning, specifically a methodology called “K-shot learning,” which involves fine-tuning the model using only K examples per stance class (favorable, against, neutral) for a new topic. This ensures balanced representation across different stances even with limited data.

To evaluate our model's adaptability, we fine-tuned it on a comprehensive set of topics, excluding six specific ones reserved for testing (detailed in Table 7 through Table 10). This approach simulates a realistic scenario where new topics arise with scarce data available.

**Table 7:** Results for Zero-shot learning.

**Topic**	**Manual topic description**				
	*F*_*favor*_	*F*_*against*_	*F*_*none*_	*F*_*avg*2_	*F*_*avg*3_
Online Learning	0.77	0.77	0.45	0.77	0.66
Neom City	0.82	0.57	0.41	0.69	0.60
Domestic Tourism	0.53	0.31	0.39	0.42	0.41
Military Conscription	0.66	0.53	0.49	0.60	0.56
Mosques Speakers	0.45	0.47	0.34	0.46	0.42
Multi Marriage	0.59	0.61	0.30	0.60	0.50
AVERAGE OVER Avg2 & Avg3				0.59	0.52

Table 7 shows the results (F1 scores) for the zero-shot learning scenario of the stance detection model using manually crafted topic descriptions. In a zero-shot setting, where the model encounters unseen topics, performance is understandably lower compared to previously trained topics. The average F1 score for both “favor” and “against” stances hovers around 0.6, indicating a basic ability to identify sentence but with less accuracy. The results for the “none” stance, representing neutral tweets, are even lower with an average F1 score of 0.34. This underscores the significant challenge the model faces in classifying neutral stances on completely new topics without any specific data for fine-tuning.

Examining individual topics, the model shows varied performance. It performed better on topics like “Online Learning” (average F1 score of 0.71), where opinions are likely more polarized. Conversely, topics such as “Domestic Tourism” and “Mosques Speakers” resulted in lower scores (average F1 score around 0.4), suggesting these topics might be more nuanced or contain more neutral language, complicating stance detection in a zero-shot scenario. Overall, the zero-shot learning results highlight the model's limitations when encountering entirely new topics. While it can still make some basic sentence predictions, the accuracy is significantly lower compared to trained topics. This emphasizes the importance of having some topic-specific data for improved performance in real-world applications.

We then employed incremental fine-tuning, progressively adapting the model with increasing amounts of data (10, 20, and 40 examples per class) for the new topics (Tables 8–10). This step-by-step approach allows us to observe the model's ability to learn from limited topic-specific data, which is crucial for real-world deployments. The significant performance improvements at the 40-shot level, with an average *F*_*avg*2_ score of 0.75, demonstrate that even a small amount of data can substantially enhance the model's effectiveness on unseen topics.

**Table 8:** Results for 10-shot learning.

**Topic**	**Manual topic description**				
	*F*_*favor*_	*F*_*against*_	*F*_*none*_	*F*_*avg*2_	*F*_*avg*3_
Online Learning	0.87	0.85	0.47	0.86	0.73
Neom City	0.83	0.66	0.45	0.74	0.64
Domestic Tourism	0.70	0.58	0.46	0.64	0.58
Military Conscription	0.69	0.71	0.42	0.70	0.61
Mosques Speakers	0.31	0.67	0.33	0.49	0.44
Multi Marriage	0.68	0.75	0.36	0.71	0.60
AVERAGE OVER Avg2 & Avg3				0.69	0.60

Table 8 shows the results (F1 scores) for stance detection on unseen topics using 10-shot learning with manually crafted topic descriptions, where the F1 score balances precision and recall to measure overall model performance. The average F1 score across all stances (“favor,” “against,” and “none") is 0.69 for *F*_*avg*2_ and 0.60 for *F*_*avg*3_, indicating moderate performance on unseen topics even with limited data. Performance varies across topics, with higher scores for “Online Learning” and “Neom City” (around 0.7) and lower scores for “Mosques Speakers” and “Military Conscription” (around 0.5), highlighting challenges in these specific domains. The model struggles more with identifying neutral stances, consistently showing lower F1 scores for “none” compared to “favor” and “against.” Overall, the results suggest that while the model can adapt to new topics with some success using 10-shot learning, there is a need for improvement in handling neutral stances and certain topic domains.

Table 9 presents the results (F1 scores) for stance detection on unseen topics using 20-shot learning with manually crafted topic descriptions, where the F1 score balances precision and recall for an overall measure of performance. The model performed well in identifying tweets expressing favorable (*F*_*favor*_) and opposing (*F*_*against*_) stances for most topics, with average F1 scores around 0.74, indicating effective learning of basic stance with limited data (20 examples per stance class). However, accurately classifying neutral tweets (“None") proved more challenging, with an average F1 score of around 0.46, highlighting difficulties in distinguishing neutral language from weakly expressed opinions on unseen topics. Performance varied across topics, with “Online Learning” and “Military Conscription” showing good performance across all stances. At the same time “Fix Domestic Tourism” and “Mosques Speakers” resulted in lower scores, particularly for the “None” stance, suggesting that topic complexity and the prevalence of neutral language influence the model's adaptability with limited data. Overall, the results demonstrate the model's potential for handling unseen topics with 20-shot learning, though improvement is needed in accurately classifying neutral stances and specific topic domains.

**Table 9:** Results for 20-shot learning.

**Topic**	**Manual topic description**				
	*F*_*favor*_	*F*_*against*_	*F*_*none*_	*F*_*avg*2_	*F*_*avg*3_
Online Learning	0.89	0.89	0.67	0.89	0.81
Neom City	0.85	0.75	0.49	0.80	0.70
Domestic Tourism	0.69	0.65	0.49	0.67	0.61
Military Conscription	0.73	0.73	0.55	0.73	0.67
Mosques Speakers	0.46	0.60	0.36	0.53	0.47
Multi Marriage	0.81	0.78	0.46	0.80	0.68
AVERAGE OVER Avg2 & Avg3				0.74	0.66

Table 10 shows the F1 scores achieved by the model using 40-shot learning with manually crafted topic descriptions. The F1 score, which balances precision and recall, provides an overall measure of model performance for each stance (“favor,” “against,” “none") on a specific topic. The average F1 scores (*F*_*avg*2_ and *F*_*avg*3_) around 0.75 indicate that the model performs well on average, effectively identifying supportive and opposing opinions in Arabic text with just 40 examples per stance class for a new topic. However, performance varies across topics. For example, topics like “Online Learning” and “Military Conscription” achieved good results across all stances, with average F1 scores above 0.7, suggesting that the model can readily learn the stance patterns associated with these topics even with limited data. Conversely, topics like “Fix Domestic Tourism” and “Mosques Speakers” proved more challenging, with lower average F1 scores, particularly for the “none” stance, indicating inherent complexity or specific challenges in identifying neutral stances in these contexts. Overall, the results are encouraging, demonstrating that the model can effectively adapt to new topics with 40 examples per stance, achieving good overall performance in stance detection for Arabic text while also highlighting the importance of considering topic-specific characteristics in real-world deployments.

**Table 10:** Results for 40-shot learning.

**Topic**	**Manual topic description**				
	*F*_*favor*_	*F*_*against*_	*F*_*none*_	*F*_*avg*2_	*F*_*avg*3_
Online Learning	0.87	0.88	0.63	0.88	0.79
Neom City	0.87	0.74	0.61	0.80	0.74
Domestic Tourism	0.72	0.66	0.51	0.69	0.63
Military Conscription	0.72	0.72	0.59	0.72	0.67
Mosques Speakers	0.49	0.76	0.45	0.62	0.57
Multi Marriage	0.81	0.79	0.49	0.80	0.70
AVERAGE OVER Avg2 & Avg3				0.75	0.68

## 6 Conclusion and discussion

This research focused on developing and evaluating a robust Arabic stance detection dataset, called ArabicStanceX, using a dataset derived from social media data. It addresses the lack of available Arabic stance detection datasets. Using the BERT architecture, we fine-tuned it to identify sentences across various topics in Arabic text.

Our exploration of different fine-tuning approaches revealed limitations with single-topic models, particularly in capturing the “none” stance and generalizing across diverse topics. In contrast, the MTSM approach showed promising results, especially when combined with manually crafted or GPT-4 generated topic descriptions.

Few-shot learning evaluations highlighted the model's potential for real-world applications, achieving good stance detection performance even with limited data (40 examples per stance class) for unseen topics. This adaptability is crucial for handling the dynamic nature of online discourse, where new topics frequently emerge.

Our findings emphasize the importance of considering topic-specific characteristics when deploying the model. Specific topics pose more significant challenges due to their complexity or the prevalence of neutral language. Future research should explore techniques to enhance performance on these nuanced topics and incorporate additional information sources beyond textual data. The results indicate that the MTSM approach, particularly with topic descriptions, holds promise for Arabic stance detection. The inclusion of topic keywords and descriptions provides the model with the necessary context for more informed predictions. Notably, manual topic descriptions were more effective than those generated by GPT-4, highlighting the potential need for human intuition in understanding nuanced topics.

However, the study has several limitations. The dataset focuses exclusively on Saudi Arabia and is sourced solely from X.com, which may restrict the generalizability of findings to other Arabic-speaking regions or platforms. Another limitation lies in class imbalance within specific topics, which may have negatively impacted the model's ability to detect minority stances. Additionally, the model struggled to handle nuanced language features such as sarcasm, implicit stances, and neutrality. Future work could expand the dataset to include other Arab countries and social media platforms, as well as explore alternative modeling approaches to better capture subtle linguistic cues. Addressing class imbalance could involve dataset resampling or data augmentation techniques.

In general, this work advances Arabic NLP by providing a foundation for effective stance detection in various topics of Arabic text. The developed model offers valuable insights into public stance and opinion dynamics within the Arabic-speaking world, with potential applications in social media analysis, market research, and other fields that rely on understanding audience perspectives. Future work should aim to improve the model's ability to detect neutral stances and enhance performance on complex and sensitive topics.

## Data Availability

The raw data supporting the conclusions of this article will be made available by the authors, without undue reservation.

## References

[B1] Al Hendi K. D. (2024). Social media addiction and usage amongst dental students in saudi arabia-a comparative study. J. Adv. Med. Dent. Sci. Res. 12, 6–13.

[B2] AlDayel A. Magdy W. (2021). Stance detection on social media: state of the art and trends. Inf. Process Manag. 58:102597. 10.1016/j.ipm.2021.102597

[B3] Alhindi T. Alabdulkarim A. Alshehri A. Abdul-Mageed M. Nakov P. (2021). Arastance: a multi-country and multi-domain dataset of arabic stance detection for fact checking. arXiv preprint arXiv:2104.13559. 10.18653/v1/2021.nlp4if-1.9

[B4] Allaway E. McKeown K. (2020). Zero-shot stance detection: a dataset and model using generalized topic representations. arXiv preprint arXiv:2010.03640. 10.18653/v1/2020.emnlp-main.717

[B5] Alturayeif N. S. Luqman H. A. Ahmed M. A. K. (2022). “Mawqif: a multi-label arabic dataset for target-specific stance detection,” in Proceedings of the Seventh Arabic Natural Language Processing Workshop (WANLP) (Stroudsburg, PA: Association for Computational Linguistics), 174–184. 10.18653/v1/2022.wanlp-1.16

[B6] Baly R. Mohtarami M. Glass J. Màrquez L. Moschitti A. Nakov P. (2018). Integrating stance detection and fact checking in a unified corpus. arXiv preprint arXiv:1804.08012. 10.18653/v1/N18-2004

[B7] Bar-Haim R. Bhattacharya I. Dinuzzo F. Saha A. Slonim N. (2017). “Stance classification of context-dependent claims,” in Proceedings of the 15th Conference of the European Chapter of the Association for Computational Linguistics: Volume 1, Long Papers, 251–261. 10.18653/v1/E17-1024

[B8] Cignarella A. T. Lai M. Bosco C. Patti V. Rosso P.. (2020). “Sardistance@ evalita2020: overview of the task on stance detection in italian tweets,” in CEUR Workshop Proceedings (Aachen: CEUR), 1–10. 10.4000/books.aaccademia.7084

[B9] Conforti C. Berndt J. Pilehvar M. T. Giannitsarou C. Toxvaerd F. Collier N. (2020). Will-they-won't-they: a very large dataset for stance detection on twitter. arXiv preprint arXiv:2005.00388. 10.18653/v1/2020.acl-main.157

[B10] Devlin J. Chang M.-W. Lee K. Toutanova K. (2018). Bert: pre-training of deep bidirectional transformers for language understanding. arXiv preprint arXiv:1810.04805. 10.48550/arXiv.1810.04805

[B11] Devlin J. Chang M.-W. Lee K. Toutanova K. (2019). “Bert: pre-training of deep bidirectional transformers for language understanding,” in Proceedings of the 2019 conference of the North American chapter of the association for computational linguistics: human language technologies, volume 1 (long and short papers) (Stroudsburg, PA: Association for Computational Linguistics), 4171–4186.

[B12] Elmadany A. Nagoudi E. M. B. Abdul-Mageed M. (2022). Orca: a challenging benchmark for arabic language understanding. arXiv preprint arXiv:2212.10758. 10.18653/v1/2023.findings-acl.609

[B13] Ferreira W. Vlachos A. (2016). “Emergent: a novel data-set for stance classification,” in Proceedings of the 2016 Conference of the North American Chapter of the Association for Computational Linguistics: Human Language Technologies (San Diego, CA: ACL). 10.18653/v1/N16-1138

[B14] Gautam A. Mathur P. Gosangi R. Mahata D. Sawhney R. Shah R. R. (2020). “#metooma: multi-aspect annotations of tweets related to the metoo movement,” in Proceedings of the International AAAI Conference on Web and Social Media, volume 14, 209–216. 10.1609/icwsm.v14i1.7292

[B15] Gorrell G. Kochkina E. Liakata M. Aker A. Zubiaga A. Bontcheva K.. (2019). “Semeval-2019 task 7: rumoureval 2019: determining rumour veracity and support for rumours,” in Proceedings of the 13th International Workshop on Semantic Evaluation: NAACL HLT 2019 (Minneapolis, MN: Association for Computational Linguistics), 845–854. 10.18653/v1/S19-2147

[B16] Grimminger L. Klinger R. (2021). Hate towards the political opponent: a twitter corpus study of the 2020 us elections on the basis of offensive speech and stance detection. arXiv preprint arXiv:2103.01664.

[B17] Hanselowski A. PVS A. Schiller B. Caspelherr F. Chaudhuri D. Meyer C. M.. (2018). A retrospective analysis of the fake news challenge stance detection task. arXiv preprint arXiv:1806.05180. 10.48550/arXiv.1806.05180

[B18] Hosseinia M. Dragut E. Mukherjee A. (2020). Stance prediction for contemporary issues: data and experiments. arXiv preprint arXiv:2006.00052. 10.18653/v1/2020.socialnlp-1.5

[B19] Jaziriyan M. M. Akbari A. Karbasi H. (2021). “ExaASC: a general target-based stance detection corpus in arabic language,” in 2021 11th International Conference on Computer Engineering and Knowledge (ICCKE) (IEEE), 424–429. 10.1109/ICCKE54056.2021.9721486

[B20] Kaur R. Sachdeva M. Kumar G. (2016). Nature inspired feature selection approach for effective intrusion detection. Indian J. Sci. Technol. 9, 1–9. 10.17485/ijst/2016/v9i42/101555

[B21] Khouja J. (2020). Stance prediction and claim verification: an arabic perspective. arXiv preprint arXiv:2005.10410. 10.18653/v1/2020.fever-1.2

[B22] Kingma D. P. Ba J. (2014). Adam: a method for stochastic optimization. arXiv preprint arXiv:1412.6980. 10.48550/arXiv.1412.6980

[B23] Lai M. Patti V. Ruffo G. Rosso P. (2020). #brexit: leave or remain? the role of user's community and diachronic evolution on stance detection. J. Intell. Fuzzy Syst. 39, 2341–2352. 10.3233/JIFS-179895

[B24] Li Y. Sosea T. Sawant A. Nair A. J. Inkpen D. Caragea C. (2021). “P-stance: a large dataset for stance detection in political domain,” in Findings of the Association for Computational Linguistics: ACL-IJCNLP 2021 (Stroudsburg, PA: Association for Computational Linguistics), 2355–2365. 10.18653/v1/2021.findings-acl.208

[B25] Mohammad S. Kiritchenko S. Sobhani P. Zhu X. Cherry C. (2016). “Semeval-2016 task 6: detecting stance in tweets,” in Proceedings of the 10th International Workshop on Semantic Evaluation (SemEval-2016) (Stroudsburg, PA: Association for Computational Linguistics), 31–41. 10.18653/v1/S16-1003

[B26] Mohammad S. M. Sobhani P. Kiritchenko S. (2017). Stance and sentiment in tweets. ACM Trans. Internet Technol. 17, 1–23. 10.1145/3003433

[B27] Reimers N. Gurevych I. (2019). Sentence-bert: sentence embeddings using siamese bert-networks. arXiv preprint arXiv:1908.10084. 10.18653/v1/D19-1410

[B28] Simsim M. T. (2011). Internet usage and user preferences in Saudi Arabia. J. King Saud Univ. Eng. Sci. 23, 101–107. 10.1016/j.jksues.2011.03.006

[B29] Sobhani P. Inkpen D. Zhu X. (2017). “A dataset for multi-target stance detection,” in Proceedings of the 15th Conference of the European Chapter of the Association for Computational Linguistics: Volume 2, Short Papers, 551–557. 10.18653/v1/E17-2088

[B30] Taulé M. Martí M. A. Rangel F. M. Rosso P. Bosco C. Patti V.. (2017). “Overview of the task on stance and gender detection in tweets on catalan independence at ibereval 2017,” in CEUR Workshop Proceedings, volume 1881 (CEUR-WS) (Stroudsburg, PA: Association for Computational Linguistics), 157–177.

[B31] Thakur K. Kumar G. (2021). Nature inspired techniques and applications in intrusion detection systems: recent progress and updated perspective. Arch. Comput. Methods Eng. 28, 2897–2919. 10.1007/s11831-020-09481-7

[B32] Vamvas J. Sennrich R. (2020). X-stance: a multilingual multi-target dataset for stance detection. arXiv preprint arXiv:2003.08385. 10.48550/arXiv.2003.08385

[B33] Wosom. (2024). Wosom. Available online at: https://wosom.ai/ (accessed May 26, 2024).

